# Prognosis and immune infiltration analysis of endoplasmic reticulum stress-related genes in bladder urothelial carcinoma

**DOI:** 10.3389/fgene.2022.965100

**Published:** 2022-09-15

**Authors:** Yaxuan Wang, Haixia Zhu, Xiaolin Wang

**Affiliations:** ^1^ Department of Medical School, Nantong University, Nantong, China; ^2^ Department of Central Laboratory, Affiliated Tumor Hospital of Nantong University & Nantong Tumor Hospital, Nantong, China; ^3^ Department of Urology, Affiliated Tumor Hospital of Nantong University & Nantong Tumor Hospital, Nantong, China

**Keywords:** BLCA, HSP90B1, immune infiltration, therapeutic target, ER stress

## Abstract

**Background:** Abnormal activation of endoplasmic reticulum (ER) stress sensors and their downstream signalling pathways is a key regulator of tumour growth, tumour metastasis and the response to chemotherapy, targeted therapy and immunotherapy. However, the study of ER stress on the immune microenvironment of bladder urothelial carcinoma (BLCA) is still insufficient.

**Methods:** Firstly, 23 ER stress genes were selected to analyse their expression differences and prognostic value in BLCA based on the existing BLCA genome atlas data. According to the expression level of ER stress-related genes in BLCA, two independent clusters were identified using consensus cluster analysis. Subsequently, the correlation between these two clusters in terms of the immune microenvironment and their prognostic value was analysed. Finally, we analysed the prognostic value of the key ER stress gene *HSP90B1* in BLCA and its corresponding mechanism that affects the immune microenvironment.

**Results:** Consensus clustering showed a worse prognosis and higher expression of immunoassay site-related genes (*HAVCR2, PDCD1, CTLA4, CD274, LAG3, TIGIT* and *PDCD1LG2*) in cluster 1 compared with cluster 2. Additionally, both TIMER and CIBERSORT algorithms showed that the expression of immune infiltrating cells in cluster 1 was significantly higher than that in cluster 2. Subsequently, HSP90B1 was identified as a key ER stress gene in BLCA, and its high expression indicated poor prognosis and was closely related to PD1. We also analysed the correlation between *HSP90B1* expression and immune-infiltrating cell related biomarkers, which showed positive results. Finally, we verified the prognostic value of *HSP90B1* in BLCA using an immunohistochemical assay in a tissue microarray of 100 patients with BLCA, validating the potential of *HSP90B1* as a prognostic biomarker in patients with BLCA.

**Conclusion:** Our work reveals that ER stress genes play a crucial role in the BLCA immunological milieu, and *HSP90B1* is a potential prognostic biomarker and therapeutic target for cancer immunotherapy.

## Introduction

Bladder urothelial carcinoma (BLCA) is a common malignant tumour of the urinary system and is the leading cause of the top ten cancer-related fatalities worldwide. In 2020, there were 81,400 new BLCA diagnoses and 17,980 BLCA-related deaths (Sung et al., 2021). Muscle-invasive BLCA and non-muscle-invasive BLCA are the two types of BLCA. More than 75% of BLCA diagnoses have a non-muscle-invasive form that can be conservatively treated locally and monitored; however, the remaining 25% have a muscle-invasive form that often necessitates cystectomy, radiation or palliative care (Chang et al., 2017). Surgical resection, chemotherapy and radiation therapy have led to considerable strides in cancer treatment, but patients’ survival times and treatment responses still vary widely. For patients with advanced BLCA and a high mutation load, immune checkpoint inhibitors (ICI) have been licensed (Zhao et al., 2019). However, the overall response rate is merely 15%–25% (Afonso et al., 2020). Thus, it is vital to develop biomarkers that can predict therapy response. The genesis and clinical and pathological symptoms of this highly heterogeneous malignant tumour vary among individuals. Furthermore, studies show that the survival and treatment of BLCA are increasingly linked to the patient’s immune system (Schneider et al., 2019). For example, cytotoxic T-lymphocyte antigen 4 (CTLA-4), programmed cell death 1 (PD1) and PD1 ligand (PD-L1) have served as essential targets for the development of new immunotherapy drugs (Qin et al., 2019). Similarly, BLCA needs to filter credible immune-related prognostic markers for better treatment responses (Kong et al., 2020).

Endoplasmic reticulum (ER) stress serves as a point of “protein quality control” in cells and facilitates several cellular functions by processing nascent membrane and secretory proteins in a Ca^2+^-dependent manner (Bettigole and Glimcher, 2015). ER stress has been shown to be a contributor to the development of a wide range of human malignancies as well as their progression to a malignant state. The fast multiplication of tumour cells is followed by an abrupt rise in the rate of protein synthesis, which always results in the activation of the unfolded protein response (UPR). As ER stress pathways impact every cancer hallmark, it is no surprise that UPR factors are prevalent in practically all cancer types (Pavlovic and Heindryckx, 2021). Poor prognosis and clinical outcome are linked to the overexpression of ER stress indicators in many different forms of cancer (Dalton et al., 2013; Matsuo et al., 2013; Chen et al., 2014; Shimizu et al., 2017). A recent study suggests that ER stress is responsible for the secretion of exosomal PD-L1 by oral squamous cell carcinoma cells and the upregulation of PD-L1 expression in macrophages, which in turn drives M2 macrophage polarization (Yuan et al., 2022). However, a thorough knowledge of ER stress in BLCA, including the interplay between ER stress regulators and the tumour immune microenvironment (TIME), is not yet available.

Recent studies have reported that HSP90B1 can regulate the growth and invasion of bladder cancer cells (Fang et al., 2019); however, its role as a prognostic biomarker of BLCA remains unexplored. In this study, a comprehensive investigation involving the expression profiles of ER stress regulators in BLCA and their connections with prognosis and involvement in TIME was conducted. In addition to this, we grouped the clusters according to the expression level of ER stress regulators, revealing a clear distinction between the two clusters in terms of tumour heterogeneity and TIME. This subgrouping helps with the risk classification and precision therapy of patients with BLCA. Subsequently, HSP90B1 was identified as a candidate for a stress regulator in the ER that is connected to immunological invasion. High levels of its expression were associated with a bad prognosis and showed a high association with PD1 in BLCA.

## Materials and methods

### Samples and datasets

The genomic data sharing (GDC) portal of the Cancer Genome Atlas (TCGA) database (https://portal.gdc.cancer.gov/) was used to obtain the clinical information of patients with BLCA. In the study, 406 BLCA tissues and 19 normal bladder samples were included. To further confirm the expression level of HSP90B1, datasets (GSE3167) from the Gene Expression Omnibus (GEO; https://www.ncbi.nlm.nih.gov/geo/) database were obtained and utilised. Additionally, 100 BLCA samples were used as the validation cohort. At Nantong Tumour Hospital, data from 100 patients with BLCA (including 41 matched normal bladder tissues) who underwent partial and radical cystectomy between June 2012 and March 2018 were also obtained. After surgery, the duration of the follow-up period ranged from one to 6 years for each patient, continuing until August 2019 (Zhu et al., 2020). Conventional written informed consent was obtained from all subjects. The ethics committee at the Nantong Tumour Hospital approved this investigation.

### Clustering analysis

Based on the expression level of selected ER stress regulators in patients with BLCA, consistency analysis using the “ConsensusClusterPlus” R package (v1.54.0), where the maximum number of clusters is six and 80 per cent of the total sample is drawn 100 times, clusterAlg = “hc”, innerLinkage = “ward.D2”, and principal component analysis (PCA) using the “ggplot2” package were performed.

### Functional analysis

Furthermore, the “Cluster Profiler” package in R was used to perform gene set enrichment analysis (GSEA), Gene Ontology (GO) and KEGG signalling pathway analyses (Wang et al., 2021).

### Correlation analysis of immune infiltration

We also employed the “immuneeconv” package in R, which incorporates various algorithms, including TIMER and CIBERSORT, to further validate the findings of our immune score assessment. In order to build the aforementioned techniques, the R Foundation for Statistical Computing (2020) version 4.0.3 was used along with “ggplot2” and “pheatmap.”

### Expression and prognostic analysis of HSP90B1 in bladder cancer

The human protein atlas database (https://www.proteinatlas.org/) was used to analyse the expression of HSP90B1 protein in BLCA and normal bladder tissues. The HSP90B1 survival was externally validated using the Kaplan–Meier plotter and PrognoScan database. Analyses of cox regression, both univariate and multivariate, were used to identify the most appropriate terms for use in the nomogram. The “forestplot” R program was used to identify the *p* value, hazard ratio (HR) and 95% confidence interval (CI) for each variable. A nomogram was constructed using the findings of a multivariate cox proportional hazards analysis to make an accurate prediction of the X-year overall recurrence. A graphical depiction of the variables that can assess the probability of recurrence for a given patient was supplied by the nomogram. This calculation was accomplished using the “rms” R package, and the points associated with each risk factor were utilized in the calculation.

### Immunohistochemistry

Samples were embedded in paraffin at a thickness of 4 nm. Deparaffinization and rehydration were performed on each slide. To eliminate aldehyde linkages from antigens, they were re-extracted using a pressure cooker and 0.01 M citrate buffer (pH 6). The slides were incubated with HSP90B1 antibody (1: 2000; ab238126, Abcam) overnight. After incubating the HRP-labelled secondary antibody for 1 h, immunodetection was performed the following day using diaminobenzidine following the manufacturer’s instructions (Yuan et al., 2021). Two independent pathologists, each of whom was blinded to the other’s clinical data, evaluated the HSP90B1 staining. A semi-quantitative immune response score (IRS) system incorporating distribution regions and staining intensities was used for the HSP90B1 staining procedure. The immunological staining intensity score ranged from 0 to 3 (0, no reaction; 1, weak response; 2, moderate response; 3, robust response). The proportions were separated into 1 (0%–25%), 2 (26%–50%), 3 (51%–75%) and 4 (76%–100%) (Zhu et al., 2020). The final score was obtained by adding the intensity score and the proportional score. The results were as follows: 0–5, low; 6–7, high.

### Statistical analysis

For statistical analyses, IBM SPSS Statistics 25 was employed. The relationships between HSP90B1 expression and clinicopathological characteristics were evaluated using the Chi-square test. This model was utilized for both univariate and multivariate assessments of prognosis. The survival curves were constructed using Kaplan–Meier analysis and log-rank testing. Furthermore, *p* values below 0.05 were regarded as significant.

## Results

### Expression divergence of ER stress genes between bladder urothelial carcinoma and adjacent normal tissues

**FIGURE 1:**
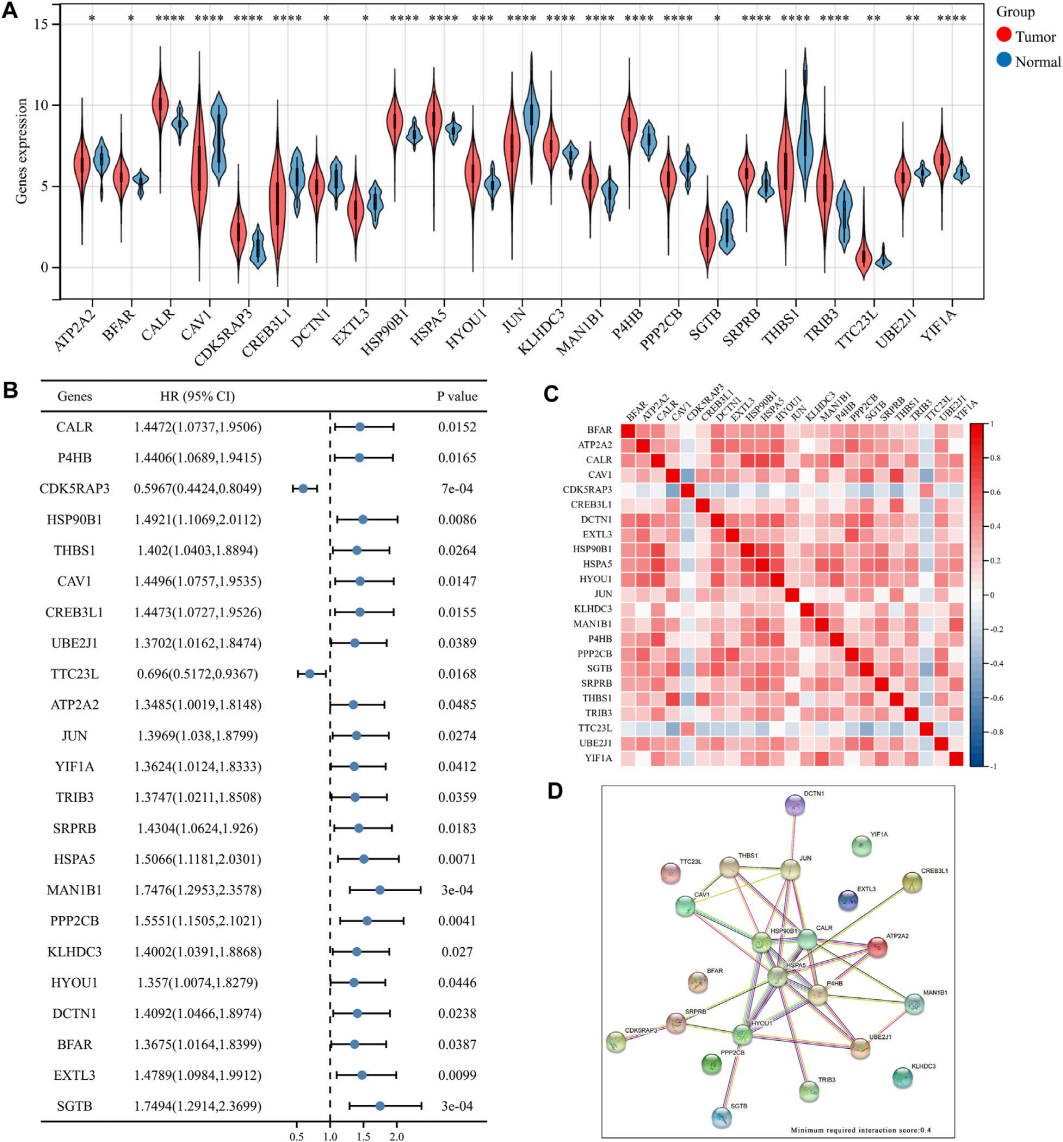
Differential expression of 23 key endoplasmic reticulum (ER) stress genes in bladder urothelial carcinoma (BLCA). **(A)** Expression of ER stress genes in BLCA and normal bladder tissues. **(B)** Overall survival forest map of 23 key ER stress genes. **(C)** Heat map of 23 key ER stress genes. **(D)** Network diagram of 23 ER stress genes.

The Molecular Signature Database v7.0 was used to download two ER stress-related gene sets (GO RESPONSE TO ENDOPLASMIC RETICULUM STRESS and GO REGULATION OF RESPONSE TO ENDOPLASMIC RETICULUM STRESS) (MSigDB,4). After removing overlapping genes, the obtained ER stress-related gene collection had 272 genes. Among these, 256 genes were discovered in the TCGA dataset (Huang et al., 2021) (Supplementary Table S1), which were screened in this study using differential expression and univariate analysis. The analyses revealed 23 key ER-related genes in BLCA. Figure 1A shows the difference in the expressions of these 23 genes between BLCA and normal tissues. The expressions of *BFAR, CALR, CDK5RAP3, HSP90B1, HSPA5, HYOU1, KLHDC3, MAN1B1, P4HB, SRPRB, TRIB3, TTC23L* and *YIF1A* in BLCA were significantly higher than that in normal tissues whereas the expressions of other genes in BLCA was lower than that in normal tissues. In Figure 1B, the forest map shows the prognostic significance of these 23 genes in the overall survival of BLCA. The low expression of CDK5RAP3 and TTC23L was significantly associated with poor overall survival and poor prognosis in patients with BLCA while the high expression of the other 21 genes was associated with the poor prognosis of BLCA. Figure 1C shows the correlation and interaction of these 23 ER stress-related genes. The results showed that *CDK5RAP3* and *TTC23L* were negatively correlated with the other 21 genes, while the other 21 genes were positively correlated. Figure 1D shows the related effects of the 23 genes using the STRING website (minimum required interaction score: 0.4), wherein the genes were mainly centred on HSPA5 and HSP90B1.

### Mutational and enrichment analysis of ER stress-related genes

**FIGURE 2:**
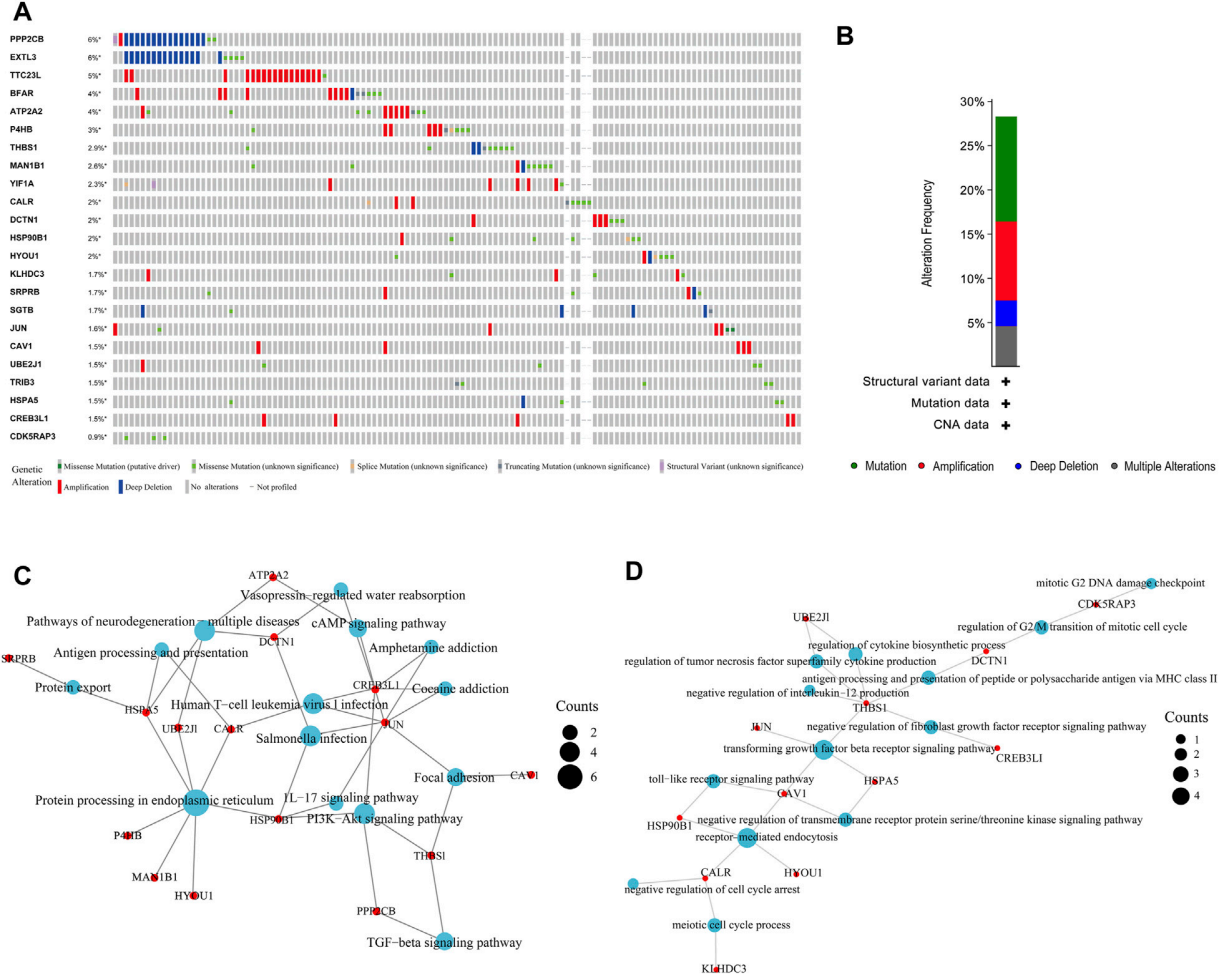
Mutation and enrichment analysis of endoplasmic reticulum (ER) stress-related genes in bladder urothelial carcinoma (BLCA). **(A, B)** Mutation analysis of ER stress-related genes in BLCA. **(C, D)** Enrichment analysis of ER stress-related genes in BLCA.

We first used the cBioPortal (https://www.cbioportal.org/) tool to analyse the mutations of these 23 genes in BLCA. Figure 2A shows that these genes have different degrees of mutations in BLCA, with *EXTL3* and *PPP2CB* having the highest mutation frequency and mainly involving deep deletion. Next, in summarising the mutation types in BLCA, the highest frequency was found to be missense mutation, followed by amplification (Figure 2B). Finally, we performed KEGG and GO enrichment analysis on these 23 ER stress-related genes. KEGG enrichment analysis revealed many signalling pathways related to immune infiltration, such as the IL17 signalling pathway, PI3K Akt signalling pathway and TGF beta signalling pathway (Figure 2C). GO enrichment analysis also showed the same results, such as the negative regulation of IL-12 production and fibroblast growth factor receiver signalling pathway (Figure 2D). All these conclusions suggest that ER stress-related genes play an important role in the immune microenvironment of BLCA.

### Cluster model of ER stress-related genes in bladder urothelial carcinoma based on consensus clustering analysis

**FIGURE 3:**
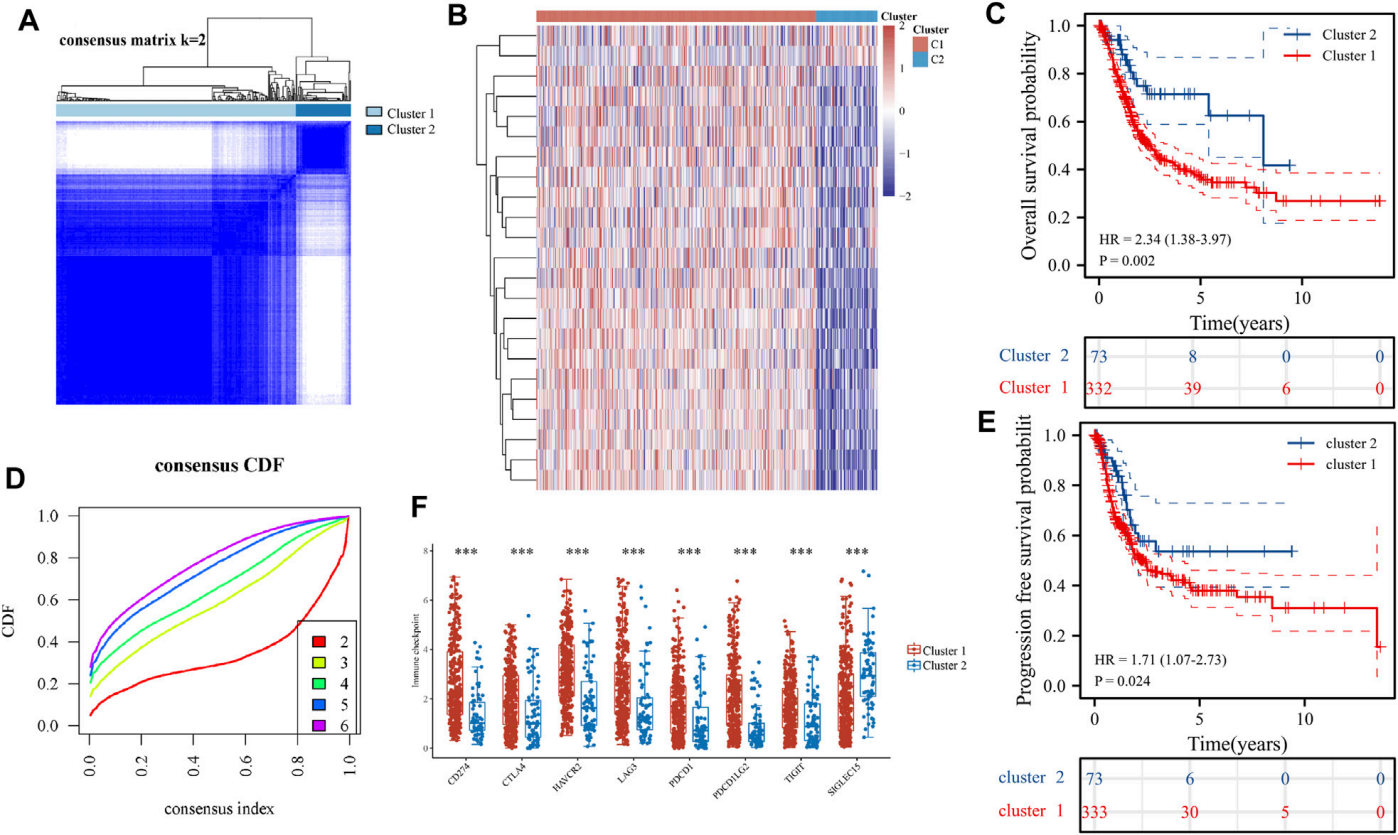
Differential expression pattern of endoplasmic reticulum (ER) stress-related genes and survival in two bladder urothelial carcinoma (BLCA) subtypes. **(A)** Consistent cluster analysis was used to split the samples of patients with BLCA into two distinct clusters. **(B)** Curves of the cumulative distribution function with k values ranging from 2 to 6. **(C)** The expression patterns of genes associated with ER stress are shown using heat maps of the two BLCA clusters. **(D)** Differential expression of immune checkpoint-related genes in two clusters. **(E, F)** Overall survival of patients with BLCA **(E)** and disease-free survival of patients with BLCA **(F)** are shown using the Kaplan–Meier curves. **(F)** The expression of genes related to immune sites between the two clusters. *p < 0.05, **p < 0.01 and ***p < 0.001.

Consensus clustering analysis of ER stress-related genes in TCGA RNA sequencing datasets was undertaken to investigate the relationship between the level of ER stress and the clinical features and prognosis of patients with BLCA. For each k, consensus matrix (CM) plots illustrate consensus values on a white to blue colour scale. The goal of CM plots is to determine the “cleanest” cluster partition where items virtually always cluster together, providing a high consensus (dark blue colour), or don’t cluster together, giving a low consensus (white) (Wilkerson and Hayes, 2010). In Figure 3A and Supplementary Figure S1A–S1D, we can clearly find that when k = 2 is the cleanest clustering partition. Furthermore, Empirical cumulative distribution function (CDF) plots display consensus distributions for each k. The “proportion of ambiguous clustering” (PAC) measure quantifies the middle segment, it is defined as the fraction of sample pairs with consensus indices falling in the interval (u1, u2) ∈ [0, 1], where u1 is a value close to 0 and u2 is a value close to 1. In permuted clustering, a low value of PAC indicates a flat middle segment and a low incidence of discordant assignments (Șenbabaoğlu et al., 2014). In Figure 3B, we can find that the curve is the flattest when k = 2 (u1 = 0.2, u2 = 0.8). So, the ideal number of clusters (k = 2) was found using CDF curves and consensus matrices, with patients being divided into two stable clusters (Huang et al., 2021) (Figure 3B). Of the 406 BLCA samples, cluster 1 included 333 samples and cluster 2 included 73 samples. The heat map revealed that there were substantial variations between these two clusters in the expression of ER stress-related genes (Figure 3C). Consequently, disparities were also found between the two clusters regarding the clinicopathological characteristics and prognosis (Supplementary Table S2). We also identified statistically significant variations in tumour stage and malignancy grade between the two clusters (*p* < 0.05) but no statistical differences were observed in age or gender (*p* > 0.05). Additionally, the expression of immune checkpoint-related genes between the two clusters was compared, which revealed significant differences between the two clusters (*p* < 0.001). Immune checkpoint blockade (ICB) has completely changed the treatment of human cancer (Jiang et al., 2018). In this study, based on the expression profile data, the tumour immune dysfunction and exclusion algorithm was used to predict the responsiveness of the two clusters to immune checkpoint inhibitors. The results show that cluster 1 was significantly better than cluster 2 (Supplementary Figure S1F). Finally, the overall survival and progression-free survival of patients in cluster 2 were significantly better than those in cluster 1 (*p* < 0.05) (Figures 3E,F). The findings suggested that the two subgroups of patients with BLCA are significantly heterogeneous. To further corroborate the results defined by the expression of ER stress regulators, we next analysed the gene expression patterns of the two clusters using PCA (Supplementary Figure S1G), which revealed significant differences in the characteristics of the two subtypes.

### Relationship between ER stress-related genes and immune cell infiltration in bladder urothelial carcinoma

**FIGURE 4:**
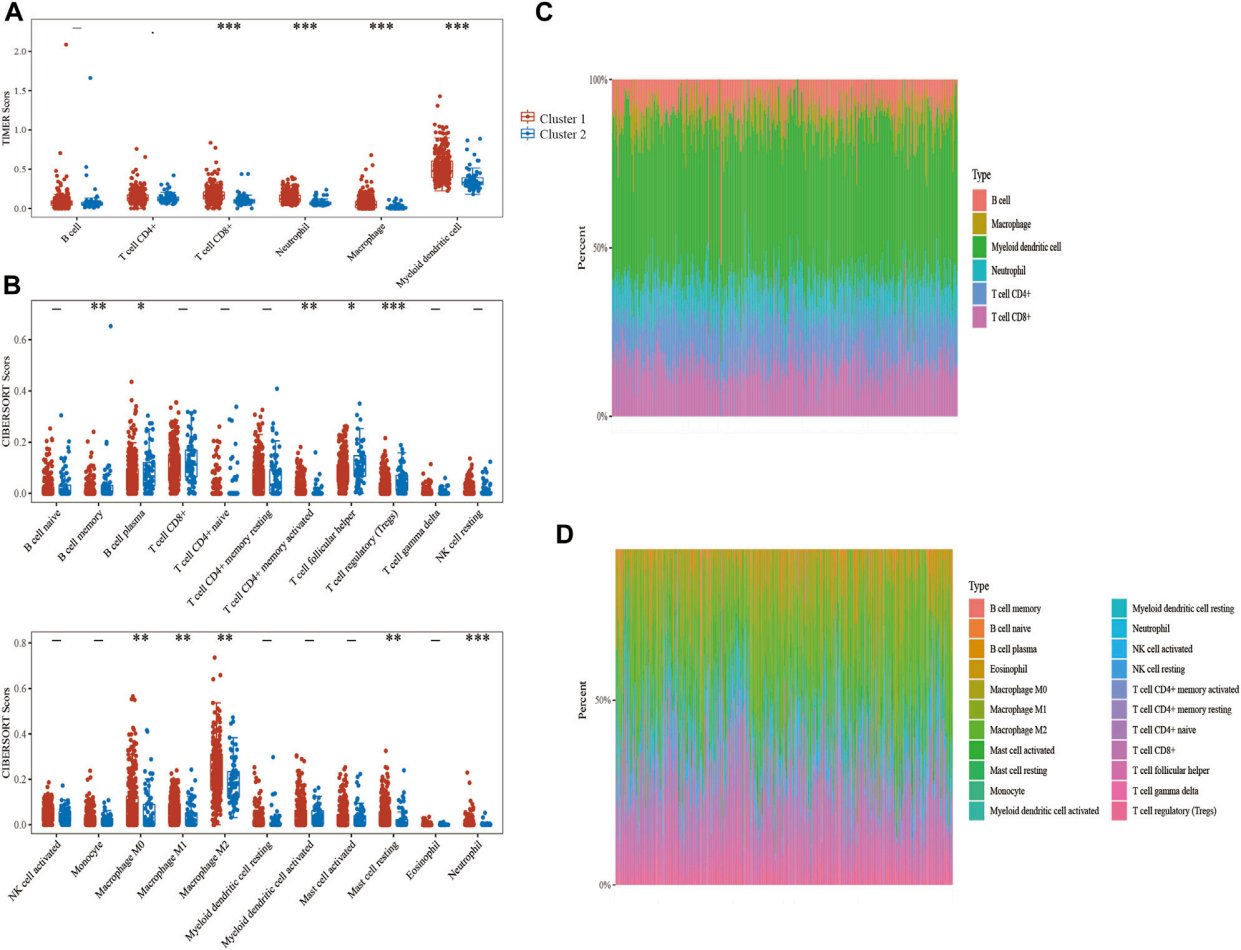
Correlation analysis between endoplasmic reticulum (ER) stress-related genes and immune infiltrating cells in bladder urothelial carcinoma (BLCA). **(A, C)** The differential expression of immune infiltrating cells and the abundance percentage of immune infiltrating cells between the two clusters was evaluated using the TIMER algorithm. **(B, D)** The differential expression of immune infiltrating cells and the abundance percentage of immune infiltrating cells between the two clusters was evaluated using the CIBERSORT algorithm.

The expression levels of the selected ER stress-related genes were used to classify the two groups, and the results showed that there were substantial variations in the immune cell infiltration of each cluster. The TIMER algorithm was used to explore the differences of many immune cell subtypes between the two clusters in BLCA samples. As shown in Figure 4A, T cell CD8^+^, neutrophil, macrophage and myeloid dendritic cells in cluster 1 significantly increased in number (*p* < 0.001), and the proportion of numerous tumour-infiltrating immune cells in the two clusters represented by the heat map was depicted as a percentage (Figure 4C). Furthermore, no significant difference was observed in the expression of B cell between the two clusters. CIBERSORT algorithm was further used to evaluate the correlation between the two clusters and T cells (Figures 4B,D). Therefore, these results suggest that ER stress-related genes have important effects on the immune microenvironment of BLCA tumours.

### Key prognostic biomarkers related to immune infiltration in ER stress-related genes in bladder urothelial carcinoma

**FIGURE 5:**
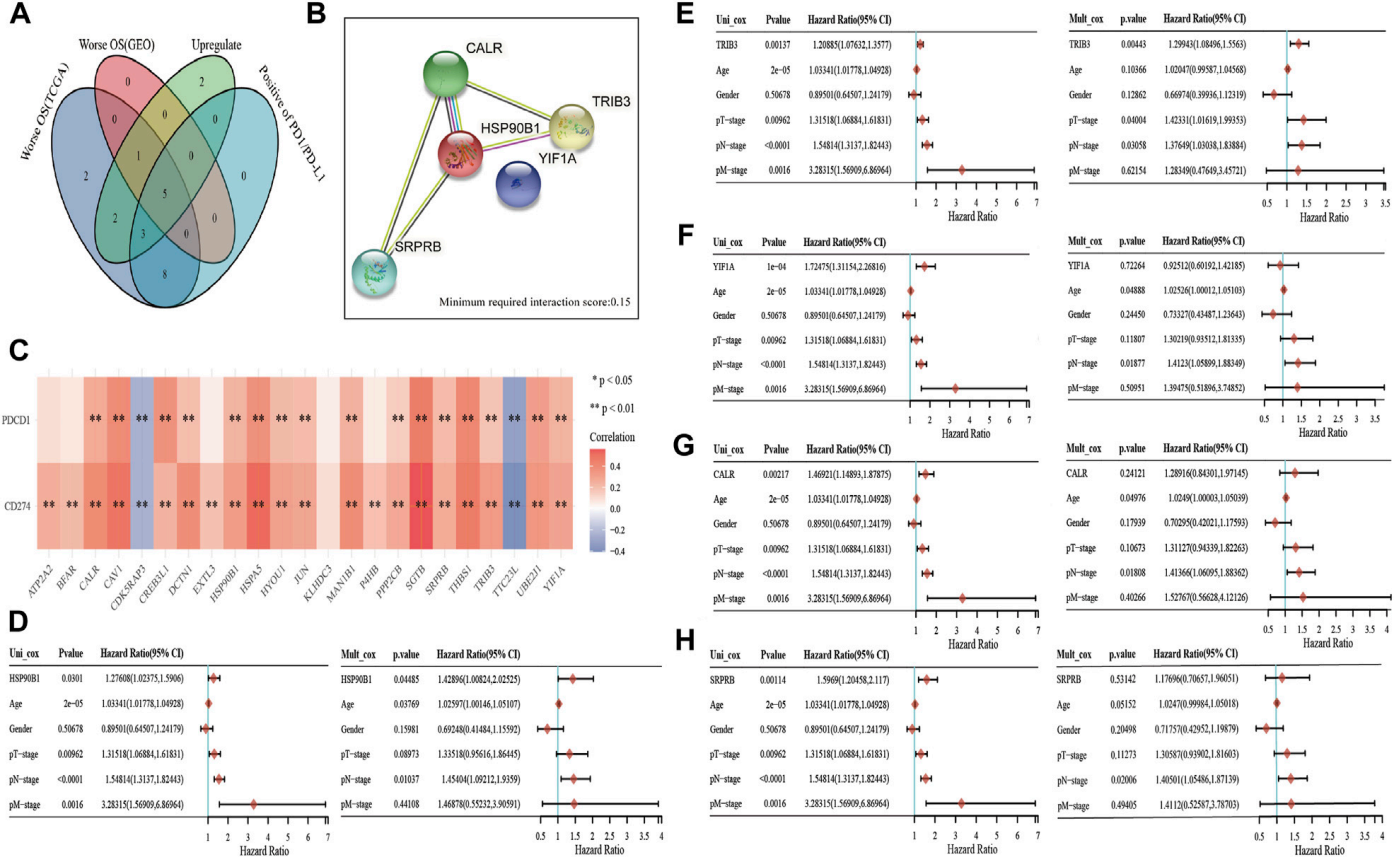
Key prognostic markers of endoplasmic reticulum (ER) stress-related genes in bladder urothelial carcinoma (BLCA). **(A)** Venn mapping is positively correlated with PD1 and PDL1, which are up-regulated key prognostic genes in BLCA. **(B)** The interaction network diagram of *CALR, HSP90B1, SRPRB, YIF1A* and *TRIB3* was analysed using the STRING database. **(C)** ER stress-related genes and PD1, PDL1 correlation heat map. **(D–H)**: The prognostic value of *CALR, HSP90B1, SRPRB, YIF1A* and *TRIB3* was analysed using univariate and multivariate analyses.

PD1 and PDL1 are the key targets of BLCA immunotherapy (Ren et al., 2022). Therefore, gene intersection analysis was used to locate key genes associated with ER stress. These genes had a significant expression level in BLCA, had a negative correlation with the prognosis of patients with BLCA in both TCGA and GSE13507 datasets (Supplementary Figure S2) and had a positive correlation with the expression of PD1 and PD-L1. Furthermore, *CALR, HSP90B1, SRPRB, YIF1A* and *TRIB3* were found to be key prognostic genes related to the immune invasion of BLCA (Figure 5A). We also used the STRING website to analyse whether these five genes interact with each other (minimum required interaction score: 0.15). The results showed that *CALR, HSP90B1, SRPRB* and *TRIB3* interacted with each other whereas HSP90B1 was at the centre (Figure 5B). PD1 and PD-L1 were also observed to be associated significantly with the expression levels of several ER stress-related genes (such as *CALR, HSP90B1, SRPRB, YIF1A* and *TRIB3*) but negatively associated with *CDK5RAP3* and *TTC23L* (Figure 5C). Finally, the association between *CALR, HSP90B1, SRPRB, YIF1A* and *TRIB3* expression and overall survival in BLCA was analysed using the Cox analysis technique; univariate analysis revealed that *HSP90B1* expression (HR = 1.27608, *p* = 0.0301), *TRIB3* expression (HR = 1.20885, *p* = 0.00137), *YIF1A* expression (HR = 1.72475, P = 1e-04), *CALR* expression (HR = 1.46921, *p* = 0.00217) and *SRPRB* expression (HR = 1.5969, *p* = 0.00114) were strongly linked with overall survival (Figures 5D–H). Additionally, multivariate analysis revealed that the expression of *HSP90B1* (*p* = 0.04485) and *TRIB3* (*p* = 0.00443) was an independent factor in determining the prognosis of patients with BLCA (Figures 5D–H).

### Clinical significance of ER stress-related key prognostic genes in bladder urothelial carcinoma

**FIGURE 6:**
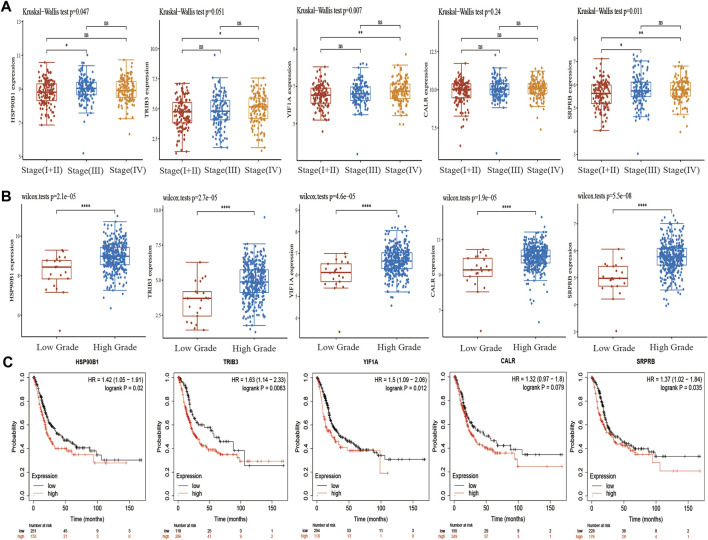
Expression and prognosis of endoplasmic reticulum (ER) stress-related genes *CALR, HSP90B1, SRPRB, YIF1A* and *TRIB3* in bladder urothelial carcinoma (BLCA). **(A)** Expression of *CALR, HSP90B1, SRPRB, YIF1A* and *TRIB3* in the staging of BLCA. **(B)** Expression of *CALR, HSP90B1, SRPRB, YIF1A* and *TRIB3* in the grading of BLCA. **(C)** Kaplan–Meier plotter was used to analyse the prognostic differences of *CALR, HSP90B1, SRPRB, YIF1A* and *TRIB3*.

In TCGA, the pathological stages in BLCA samples were divided into four stages, including 2 samples in stage I, 130 samples in stage II, 140 samples in stage III and 134 samples in stage IV. We first analysed the differential expression of *CALR, HSP90B1, SRPRB, YIF1A* and *TRIB3* in the various pathological stages. The results showed that there were significant differences among *HSP90B1, YIF1A* and *SRPRB* in the pathological stages (*p* < 0.05) but no significant difference was observed between *TRIB3* and *CALR* (*p* > 0.05) (Figure 6A) Subsequently, we also analysed the differential expression of *CALR, HSP90B1, SRPRB, YIF1A* and *TRIB3* in high-grade BLCA (n = 384) and low-grade BLCA (n = 21) samples. *CALR, HSP90B1, SRPRB, YIF1A* and *TRIB3* were up-regulated in high-grade BLCA samples (*p* < 0.001). The prognostic significance of *HSP90B1, SRPRB, YIF1A* and *TRIB3* was also confirmed in an independent BLCA cohort using Kaplan–Meier plotters (*p* < 0.05) however, that of *CALR* was not confirmed (*p* > 0.05). Subsequently, *HSP90B1* was observed to be the only gene that could predict the prognosis of BLCA and showed significant differences in pathological stages and grades. Thus, *HSP90B1* is an important ER stress-related gene associated with the prognosis and immune infiltration of BLCA.

### High *HSP90B1* expression is an independent prognostic biomarker in bladder urothelial carcinoma

**FIGURE 7:**
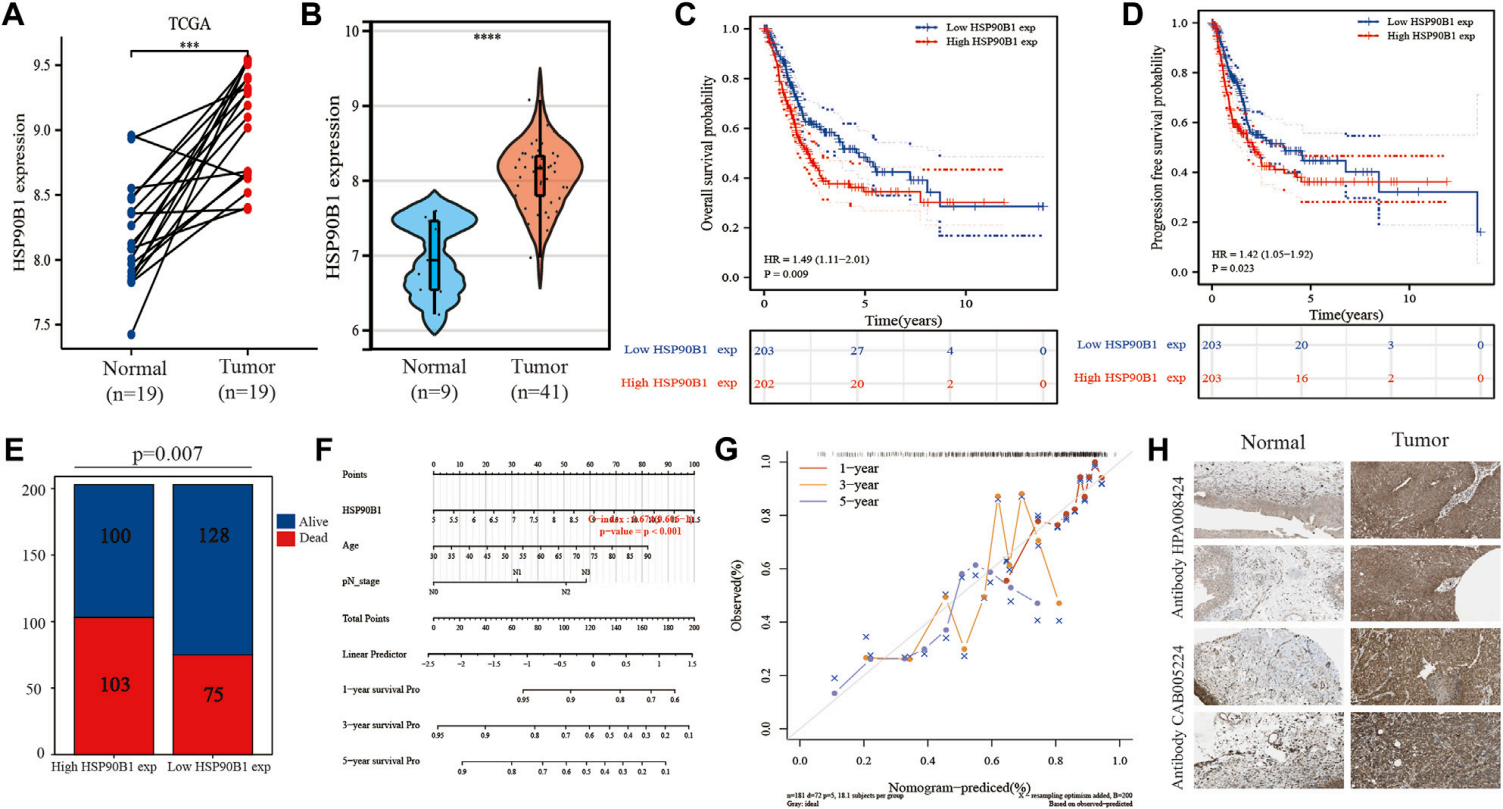
Expression and prognosis of endoplasmic reticulum (ER) stress-related gene *HSP90B1* in bladder urothelial carcinoma (BLCA). **(A)** Expression of *HSP90B1* in paired BLCA samples. **(B)** Expression of *HSP90B1* in BLCA and normal tissues in the GSE3167 dataset. C, **(D)** Prognostic significance of *HSP90B1* expression in overall survival and progression-free survival of BLCA. **(E)** Comparison of death/survival ratio between high *HSP90B1* expression group and low *HSP90B1* expression group. **(F, G)** The nomogram and calibration curve were drawn based on the results of multivariate Cox regression analysis. **(H)** Expression of HSP90B1 protein in BLCA.

Figure 1 shows that there is a significant difference in the expression of *HSP90B1* between BLCA and adjacent normal tissues. In the paired BLCA samples (n = 19) in the TCGA database, there was a significant difference in the amount of *HSP90B1* expression between BLCA and normal bladder tissues (*p* < 0.001) (Figure 7A). Additionally, we evaluated the expression data from the GEO database to provide a more in-depth illustration of the expression of *HSP90B1* in BLCA. According to the findings, *HSP90B1* was significantly overexpressed in BLCA compared to normal tissues in the GSE3167 datasets (Figure 7B). In addition, the prognostic significance of upregulated *HSP90B1* showed a worse overall survival and progression-free survival than the downregulated group (Figures 7C,D). High *HSP90B1* expression in patients with BLCA was associated with a reduced percentage of alive and dead patients, as seen by a stacked bar chart. This was in contrast to patients with BLCA who had a low level of *HSP90B1* expression (*p* = 0.007) (Figure 7E). In Figure 5D, multivariate Cox regression analysis showed that *HSP90B1* expression, age and N stage could be used as prognostic indicators for patients with BLCA. Based on this, we drew the calibration curves of the nomogram and overall survival nomogram models (Figures 7F,G). Finally, we analysed the expression level of HSP90B1 protein in four pairs of BLCA tissues and adjacent normal tissues by The Human Protein Atlas website. We found that the staining intensity of HSP90B1 protein in BLCA tissues was significantly higher than that in normal bladder tissues. We found that the staining intensity of HSP90B1 protein in BLCA tissues was significantly higher than that in normal bladder tissues.

### Verification of HSP90B1 expression and its prognosis in bladder urothelial carcinoma

**FIGURE 8:**
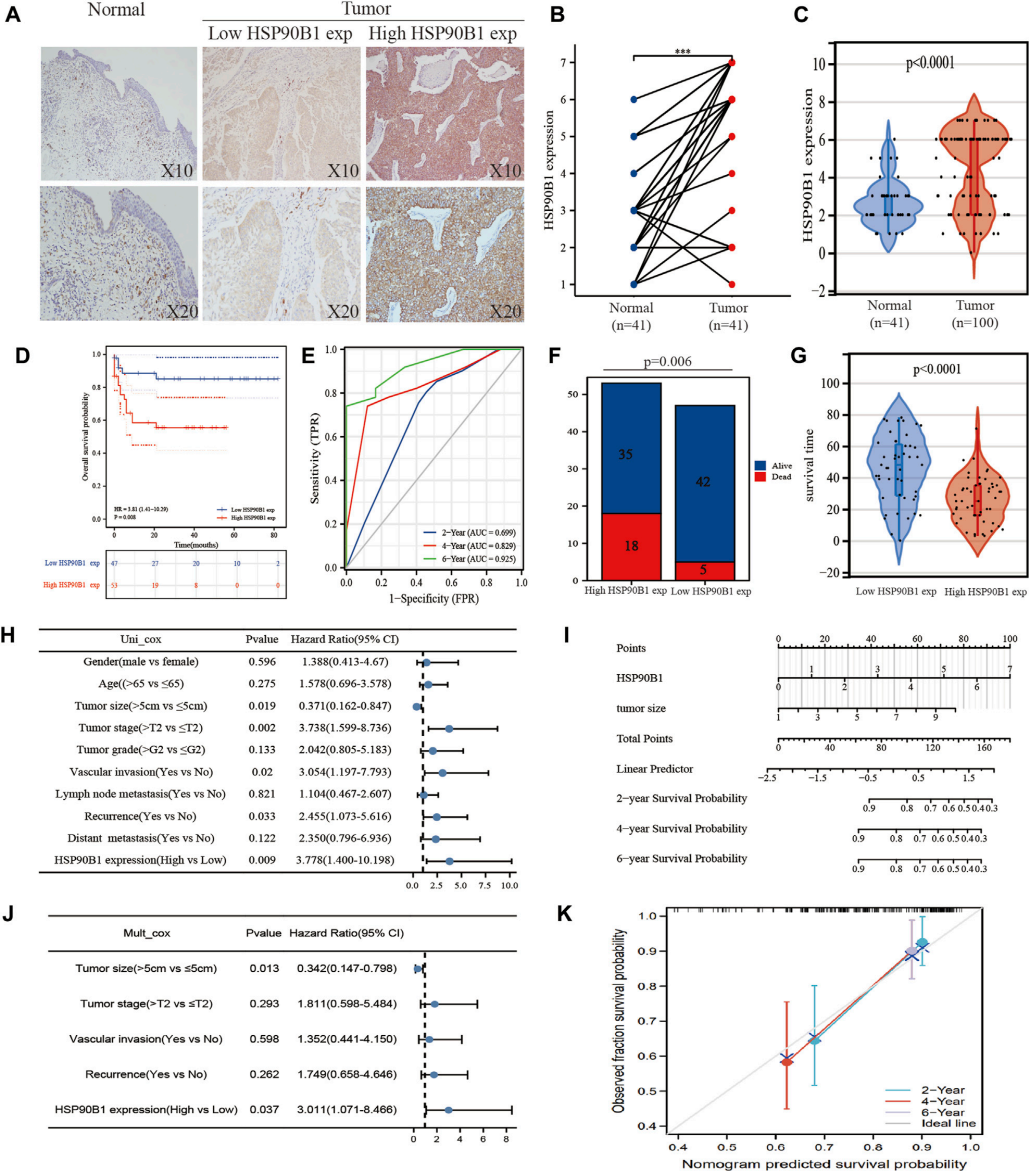
To verify the expression of HSP90B1 in bladder urothelial carcinoma (BLCA) and its prognostic significance. **(A)** The expression of HSP90B1 protein in BLCA was detected using immunohistochemistry. **(B)** HSP90B1 protein was expressed in paired samples of BLCA. **(C)** HSP90B1 protein was expressed in unmatched samples of BLCA. D, **(E)** Prognostic difference and receiver operating characteristic curve of overall survival in BLCA between the HSP90B1 high expression group and HSP90B1 low expression group. **(F)** Difference analysis of survival and death rate between the high and low HSP90B1 expression groups in patients with BLCA. **(G)** Analysis of the difference in survival time between the HSP90B1 high expression group and HSP90B1 low expression group in patients with BLCA. **(H, J)** Univariate and multivariate cox regression analyses of HSP90B1 and pathological parameters on the prognosis of BLCA. **(I, K)** Nomograms and calibration curves were drawn to predict the impact of HSP90B1 and tumour size on the 2, 4 and 6-years prognosis of patients with BLCA.

To verify the results obtained from the TCGA and GEO databases, we detected the expression and prognosis of HSP90B1 in 100 patients with BLCA and 41 matched normal bladder tissues. Immunohistochemical results showed that the expression of HSP90B1 in BLCA was significantly higher than that in normal bladder tissues (Figure 8A). Figures 8B,C show that the expression of HSP90B1 in paired BLCA samples and unmatched BLCA samples was significantly higher than that in normal bladder tissues (*p* < 0.001). The survival curve and receiver operating characteristic curve were drawn according to HSP90B1 expression, survival time and survival status. The results showed that the prognosis of patients with BLCA having a high HSP90B1 expression was poor (Figure 8D). Additionally, HSP90B1 showed a strong predictive capacity, as the area under the curve values of HSP90B1 expression for predicting 2, 4 and 6-years survival were 0.69, 0.82 and 0.925, respectively (Figure 8E). A stacked bar chart and a violin plot (Figures 8F,G) demonstrated that the alive/dead ratio and survival time of patients with BLCA having a high HSP90B1 expression was lower than those with low HSP90B1 expression. Additionally, the survival time of patients with high HSP90B1 expression was shorter than those with low HSP90B1 expression (*p* < 0.05). Furthermore, univariate analysis revealed that tumour size (HR = 0.370, *p* = 0.019), tumour stage (HR = 3.738, *p* = 00,002), vasculature invasion (HR = 3.054, *p* = 0.02) and recurrence (HR = 2.455, *p* = 0.033) were all associated with overall survival (Figure 8H). A multivariate analysis (Figure 8J) also showed that tumour size (*p* = 0.013) and HSP90B1 expression (*p* = 0.037) were independent factors for prognosis in patients with BLCA. In Supplementary Table S3, the HSP90B1 high expression group and HSP90B1 low expression group show significant differences in BLCA stage, grade, vascular invasion and lymph node metastasis (*p* < 0.05). Similarly, the calibration curves of the nomogram and overall survival nomogram models were constructed (Figures 8I,K).

### Correlation between *HSP90B1* and the immune microenvironment in bladder urothelial carcinoma

**FIGURE 9:**
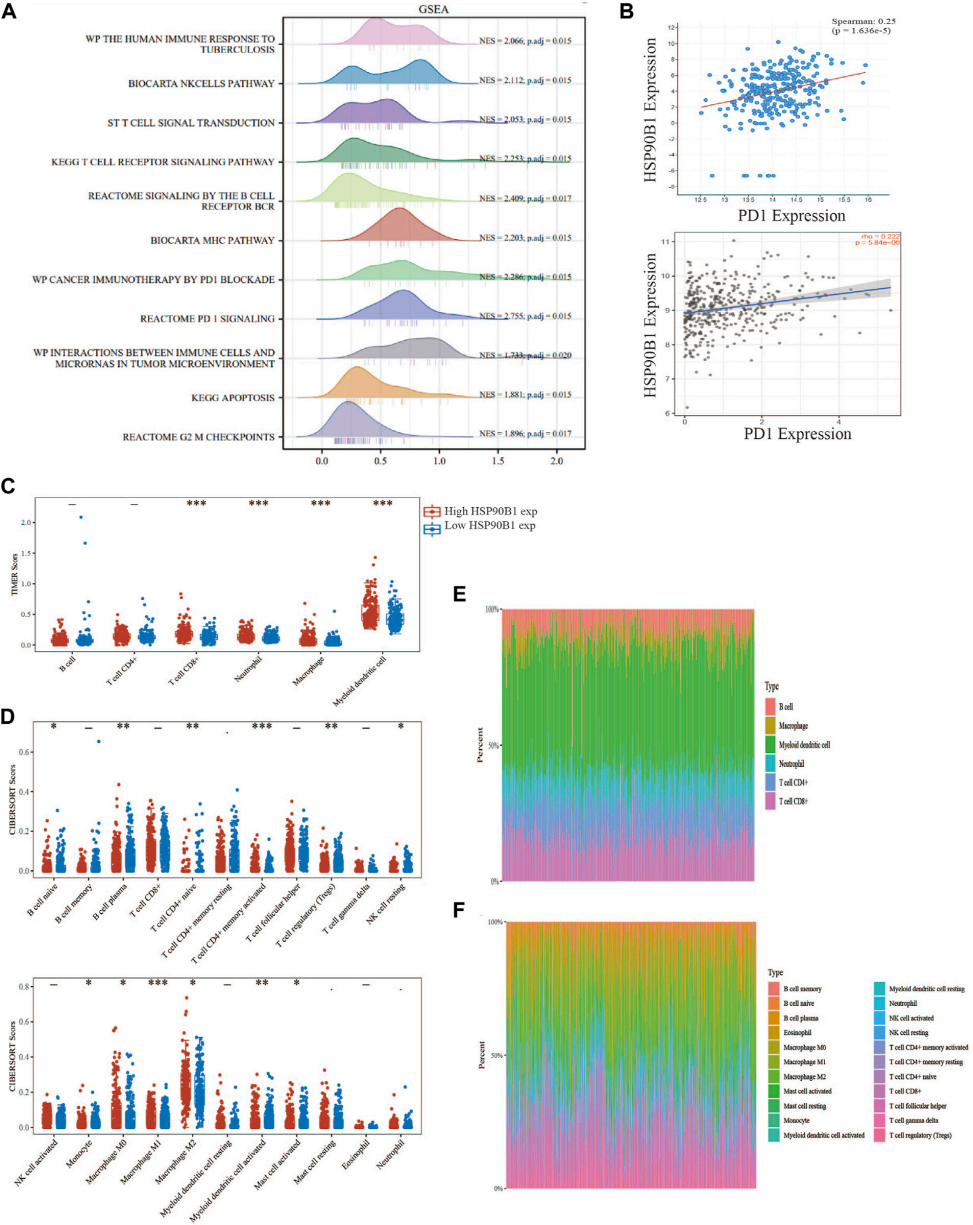
*HSP90B1* plays an important role in the immune microenvironment of bladder urothelial carcinoma (BLCA). **(A)** The high and low expression of *HSP90B1* were grouped for gene set enrichment analysis. **(B)** Correlation analysis between *HSP90B1* and PD1. **(C, E)** TIMER algorithm was used to evaluate the correlation between *HSP90B1* and immune infiltrating cells. **(D, F)** CIBERSORT algorithm was used to evaluate the correlation between *HSP90B1* and immune infiltrating cells.

GSEA was performed by grouping the *HSP90B1* high expression and low expression groups. The results showed that *HSP90B1* was significantly correlated with the immune microenvironment in BLCA, including “The human immune response to tuberculosis,” “T cell signal transduction,” “PD1 signalling” and “Cancer immunotherapy by PD1 blockade.” Enrichment analysis also showed that *HSP90B1* was closely related to PD1, which was used as a prognostic marker for immunotherapy in BLCA. Therefore, further analysis of the correlation between *HSP90B1* and PD1 in BLCA may help us better understand the potential of *HSP90B1* in BLCA immunotherapy (Figure 9A). Subsequently, we found a positive correlation between *HSP90B1* and PD1 through TIMER2.0 and cBioPortal website analysis (*p* < 0.05) (Figure 9B). Subsequently, we evaluated the expression difference of immune cell subtypes and the abundance percentage of immune infiltrating cells between the *HSP90B1* high expression group and *HSP90B1* low expression group through TIMER and CIBERSORT algorithm, revealing a positive correlation (Figures 9C–F). We also found a significant correlation between *HSP90B1* and most of the biomarkers of immune infiltrating cells (Supplementary Table S4). Finally, we explored the correlation between *HSP90B1* and immunosuppressants and immunoagonists using the TISIDB website (http://cis.hku.hk/TISIDB/index.php) (Supplementary Figure S3). Therefore, these findings suggest that *HSP90B1* plays an important role in the immune microenvironment of BLCA.

## Discussion

BLCA, the most prevalent kind of genitourinary cancer, has a high prevalence and an extremely high incidence worldwide. The management of BLCA has been evolving not only by advancements in traditional therapies, such as surgery and chemotherapy but also by the introduction of immunotherapeutic techniques. This is in addition to the early identification via cytology, which is also a recent advancement (Abd El-Salam et al., 2022). However, identifying more precise and individualized ways to treat muscle-invasive BLCA has become a popular research topic despite recent advancements in therapies, such as local or systemic immunotherapy, chemotherapy and radiation (Nie et al., 2021). Evidence is mounting that tumour cells may create and interact with the tumour microenvironment, reprogramming and controlling tumour progression, metastasis and treatment response. Moreover, anti-cancer treatment, including immunotherapy, is speculated to be hampered by ER stress (Gao et al., 2015). However, the potential role of ER stress in the BLCA immune microenvironment remains elusive. In this study, consensus clustering of selected ER stress regulators allowed for the identification of two distinct subgroups with distinct clinical characteristics, prognoses and TIME. Among these, *HSP90B1* was identified as the potential immune infiltration-related ER stress regulator. HSP90B1 is increased under a variety of stress situations that disrupt ER equilibrium (Ansa-Addo et al., 2016). HSP90B1 regulates the balance between cancer cell survival and death by maintaining ER protein folding capacity, ER stress sensors, and suppressing ER-associated pro-apoptotic machinery (Duan et al., 2021). Unfortunately, the significant role of *HSP90B1* gene in BLCA has not been analysed from the perspective of ER stress. However, in our study, gene enrichment analysis showed that the ER stress gene *HSP90B1* could affect apoptosis and cell cycle of bladder cancer cells. This conclusion provides a basis for further exploration of how the *HSP90B1* gene affects ER stress in BLCA.

The ER is a distinctive intracellular membrane structure that is involved in various biological processes, such as biosynthesis, lipid metabolism and calcium homeostasis. Additionally, it is responsible for the folding and secretion of more than 30% of the proteins that are found within the cell. Crosstalk with other organelles, such as the mitochondria, lysosomes, Golgi apparatus and nucleus, also allows it to transduce diverse signals and stressors (Oakes, 2017; Wu et al., 2021). Multiple cellular stressors, both internal and external, alter intracellular protein homeostasis in tumour cells. ER, as the key organelle for protein quality control, is responsible for protein homeostasis via precise processes, including UPR, protein clearance via ER-associated degradation and autophagy, which contributes to tumour development, metastasis, angiogenesis and chemoradiotherapy resistance (Bi et al., 2005; Wu et al., 2015; Preston and Brodsky, 2017; Chen et al., 2021). Many studies have reported that ER stress closely regulates the proliferation of BLCA cells. For example, Derlin-1, also known as ER-related degradation protein-1, is a protein that is essential to the ER degradation pathway and can interact with a wide range of other proteins. Derlin-1 is capable of forming a protein complex with ubiquitin ligase, ubiquitin protein and p97, which contains valine, and subsequently neutralizes the ER stress by cooperating with the major histocompatibility complex I to co-regulate substrate protein and promote the destruction of unfolded or misfolded proteins (Iida et al., 2011; Christianson and Ye, 2014; Mehnert et al., 2014). Studies also report that Derlin-1 expression in BLCA tissue is higher than that in normal adjacent tissues and is associated with tumour stage, histological grade, lymph node involvement and muscle invasiveness (Wu et al., 2016). Additionally, studies also report that extracellular vesicles from BLCA cells could stimulate the UPR during ER stress and inflammation and promote BLCA proliferation, development and recurrence (Wu et al., 2019). Inhibiting protein synthesis and promoting unfolded protein breakdown are two additional ways that UPR might reduce ER stress. If the adaptive systems’ capacity for stress is exceeded, cells will undergo apoptosis through a variety of TUPR-mediated pathways (Wang et al., 2022). In conclusion, these studies indicate that ER stress plays an important role in BLCA.

Recent advances in precision medicine have had a significant impact on how human malignancies are treated, highlighting the importance of identifying more precise subtypes of various diseases using multiple biological and clinical factors (Dupont et al., 2021). Increasingly studies on the groupings of patients with tumours based on their genetic spectrum, each with distinct phenotypes, prognoses and therapeutic responses, have been reported (Tan et al., 2019). In our study, patients with BLCA were divided into two clusters based on the expression features of ER stress-related genes using consensus cluster analysis. The expression of ER stress-related genes varied considerably between the two clusters, and the prognosis of cluster 1 patients was much poorer than that of cluster 2 patients. Subsequently, further analysis showed that the expression of *CD274, PDCD1, CTLA4, HAVCR2, LAG3, PDCD1LG2* and *TIGIT* in cluster 1 was significantly higher than that in cluster 2, whereas that of *SIGLEC15* in cluster 2 was significantly higher than that in cluster 1. Moreover, the prognosis of cluster 1 was significantly worse than that of cluster 2, which could be attributed to the above differential expression. Additionally, both the TIMER and CIBERSORT algorithms showed that the immune infiltrating cells had higher expression levels in cluster 1 with poor survival prognosis. Finally, *HSP90B1* was identified as a key prognostic gene of ER stress in BLCA and was associated with immune infiltration, indicating its potential as a biomarker to predict the prognosis of patients with BLCA. In the subsequent GSEA, the high and low expression of *HSP90B1* was used as a grouping factor, revealing more enriched immune-related pathways. Notably, the “PD1 pathway” and “Cancer immunotherapy by PD1 blockade” were directly enriched. This indicated that there is a high correlation between *HSP90B1* and PD1 and also provides a basis for the potential of *HSP90B1* in BLCA immunotherapy. Unfortunately, the mechanism of H*SP90B1* regulating the immune microenvironment of BLCA could not be elucidated in this study. However, the results of GSEA also include the apoptosis signalling pathway, which could be a potential mechanism of *HSP90B1* regulating the immune microenvironment of BLCA; however, further experiments are required to verify this conclusion.

## Conclusion

In conclusion, this research comprehensively examined the expression profile of ER stress-related genes in BLCA and its association with prognosis and TIME. Consensus clustering of ER stress-related genes identified two distinct subgroups of BLCA having distinct tumour heterogeneity and TIME. This subgrouping could aid in the risk classification and personalized treatment of patients with BLCA. *HSP90B1* was shown to be an independent predictor of prognosis in patients with BLCA and was found to be associated with PD1 expression among the chosen ER stress-related genes. However, further studies are required to verify the important role of *HSP90B1* in the immune microenvironment of BLCA.

## Data Availability

The datasets presented in this study can be found in online repositories. The names of the repository/repositories and accession number(s) can be found in the article/Supplementary Material.
