# Supplementary material for: Prognosis and immune infiltration analysis of endoplasmic reticulum stress-related genes in bladder urothelial carcinoma
Source: Front Genet. 2022 Sep 15;13:965100. doi: 10.3389/fgene.2022.965100 (PMC9520708; doi:10.3389/fgene.2022.965100)
Supplement: Supplementary file 1 [file DataSheet2.docx]

**Supplementary Figure**


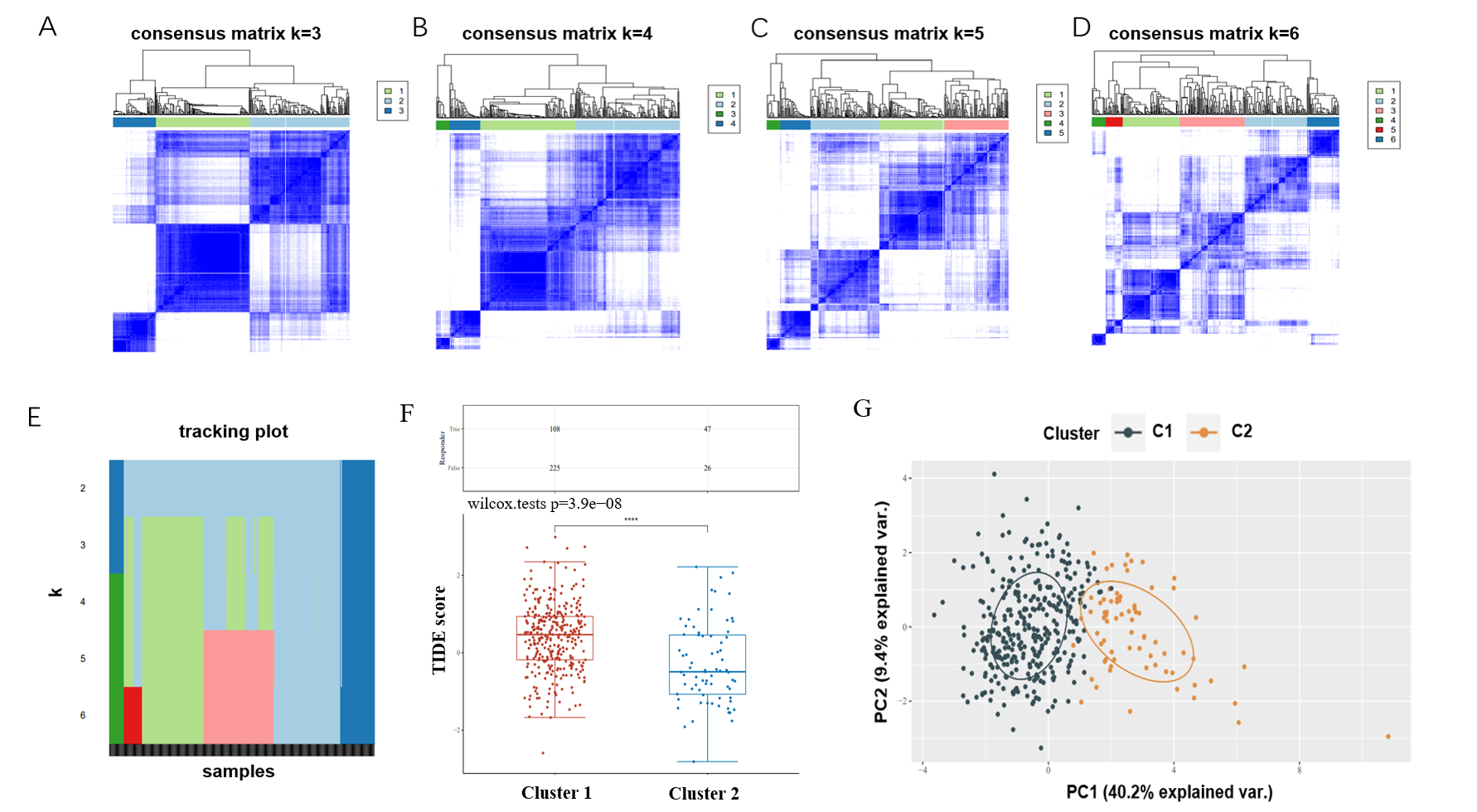


**Supplementary Figure 1. Consensus clustering analysis of endoplasmic reticulum (ER) stress-related genes.**


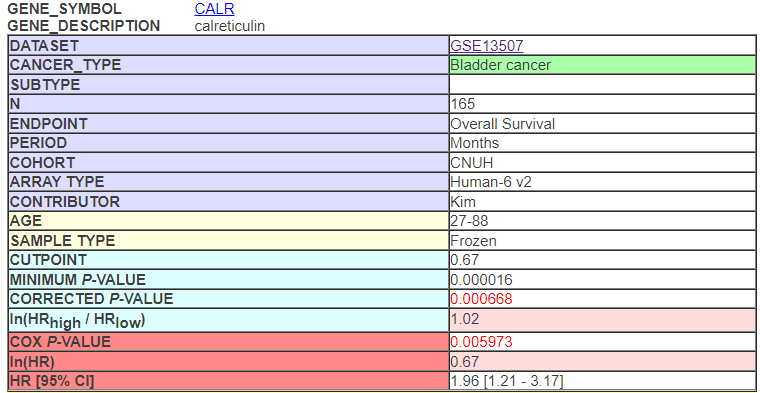

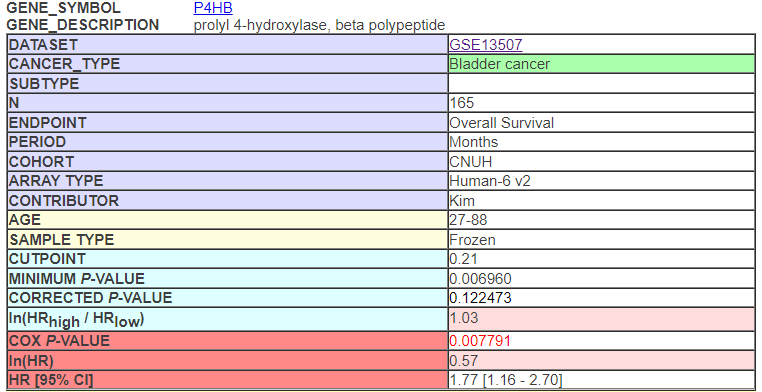


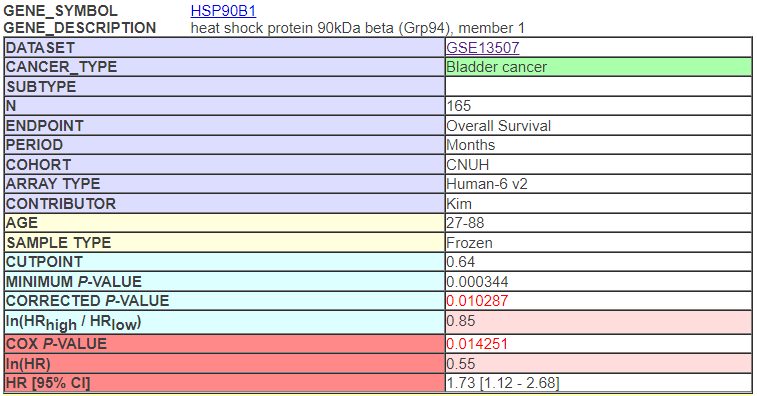


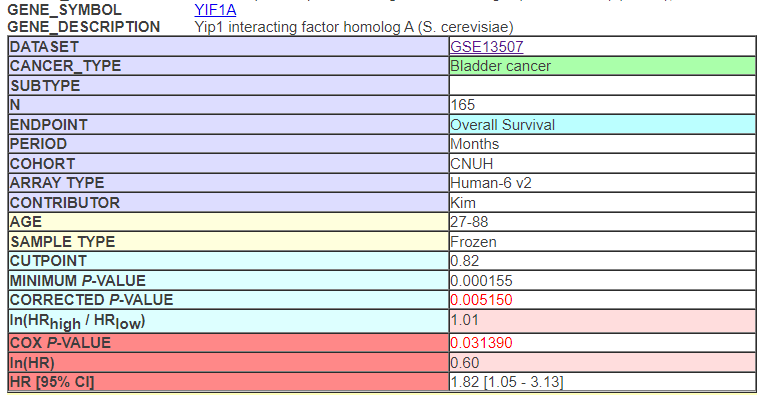


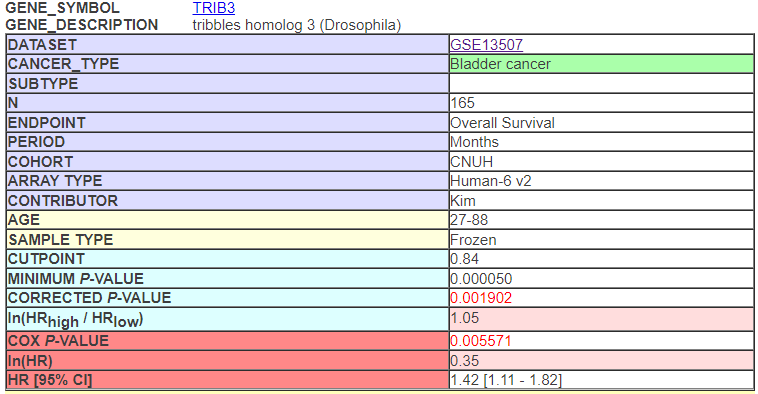


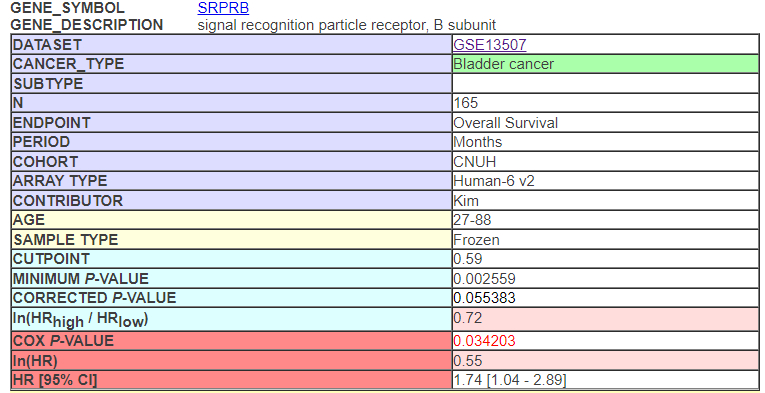


**Supplementary Figure 2.** **Prognostic endoplasmic reticulum (ER) stress genes in the GSE13507 dataset.**


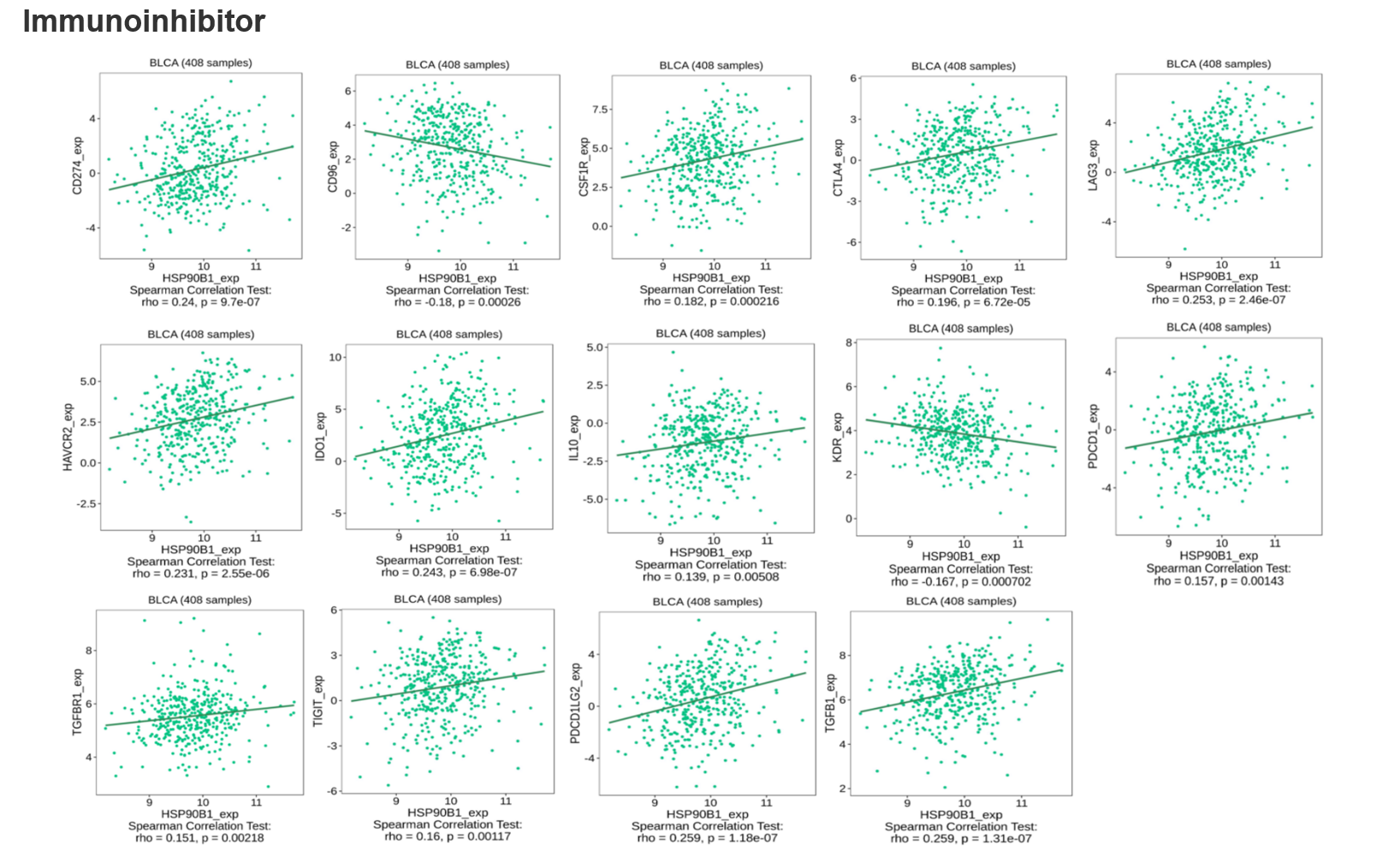

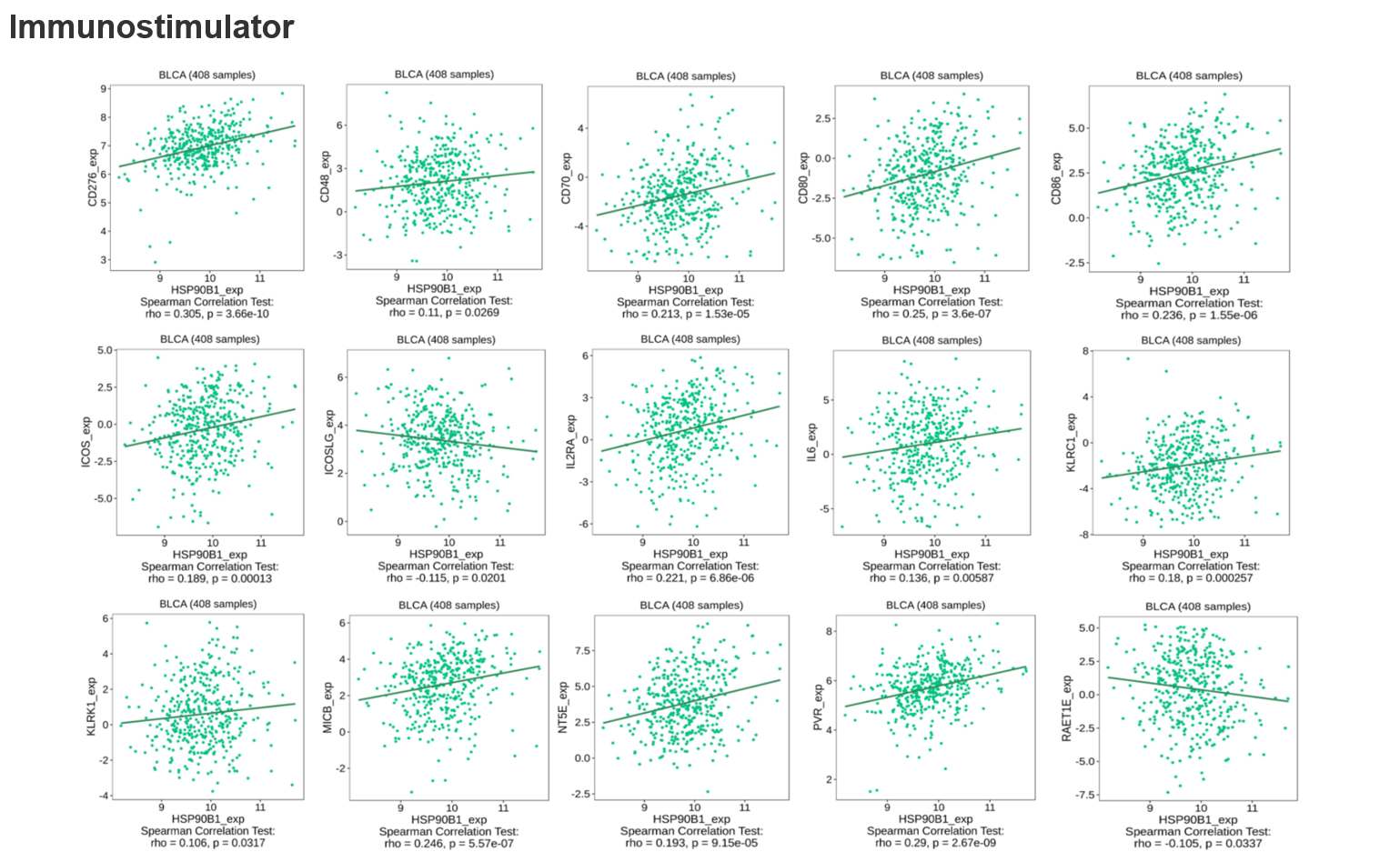

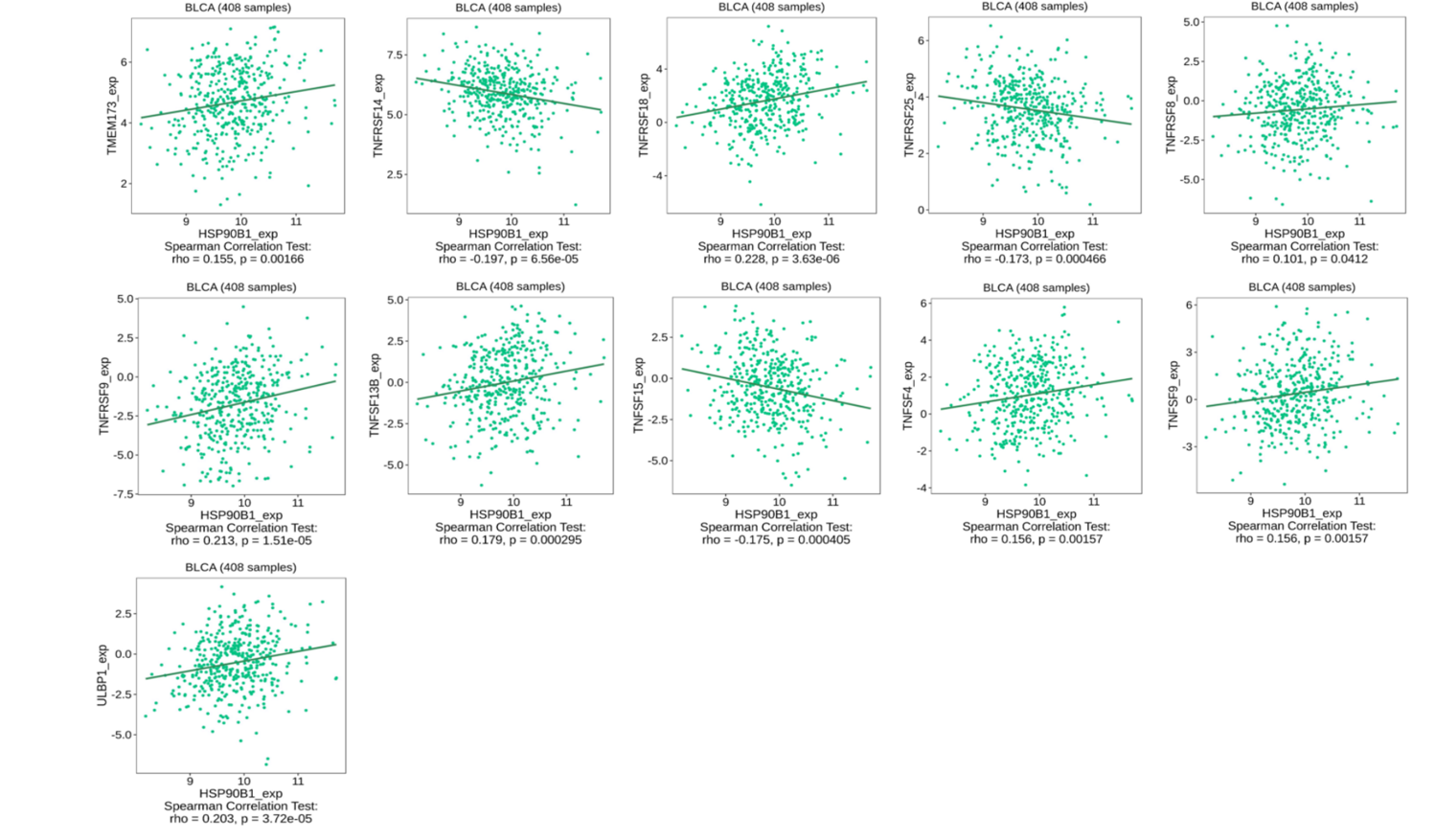


**Supplementary Figure 3.** **The correlation between HSP90B1 and immunosuppressants and immunoagonists.**
